# The Mismatch Between Bony Endplates and Grafted Bone Increases Screw Loosening Risk for OLIF Patients With ALSR Fixation Biomechanically

**DOI:** 10.3389/fbioe.2022.862951

**Published:** 2022-04-08

**Authors:** Jing-Chi Li, Tian-Hang Xie, Zhuang Zhang, Zhe-Tao Song, Yue-Ming Song, Jian-Cheng Zeng

**Affiliations:** ^1^ Department of Orthopedic Surgery and Orthopedic Research Institute, West China Hospital/West China School of Medicine for Sichuan University, Chengdu, China; ^2^ Department of Imaging, West China Hospital, Chengdu, China

**Keywords:** oblique lumbar interbody fusion, grafted bony occupancy rate, screw loosening, biomechanical deterioration, anterior lateral single rod fixation

## Abstract

The mismatch between bony endplates (BEPs) and grafted bone (GB) triggers several complications biomechanically. However, no published study has identified whether this factor increases the risk of screw loosening by deteriorating the local stress levels. This study aimed to illustrate the biomechanical effects of the mismatch between BEP and GB and the related risk of screw loosening. In this study, radiographic and demographic data of 56 patients treated by single segment oblique lumbar interbody fusion (OLIF) with anterior lateral single rod (ALSR) fixation were collected retrospectively, and the match sufficiency between BEP and GB was measured and presented as the grafted bony occupancy rate (GBOR). Data in patients with and without screw loosening were compared; regression analyses identified independent risk factors. OLIF with different GBORs was simulated in a previously constructed and validated lumbosacral model, and biomechanical indicators related to screw loosening were computed in surgical models. The radiographic review and numerical simulations showed that the coronal plane’s GBOR was significantly lower in screw loosening patients both in the cranial and caudal vertebral bodies; the decrease in the coronal plane’s GBOR has been proven to be an independent risk factor for screw loosening. In addition, numerical mechanical simulations showed that the poor match between BEP and GB will lead to stress concentration on both screws and bone-screw interfaces. Therefore, we can conclude that the mismatch between the BEP and GB will increase the risk of screw loosening by deteriorating local stress levels, and the increase in the GBOR by modifying the OLIF cage’s design may be an effective method to optimize the patient’s prognosis.

## Introduction

The anterior lateral single rod (ALSR) fixation system can reconstruct instant postoperative stability in a single incision for oblique lumbar interbody fusion (OLIF) patients. As a hardware-related complication, screw loosening has been widely reported, negatively affecting patients’ rehabilitation and deteriorating long-term prognosis (Bokov et al., 2019; Zou et al., 2020). The deterioration of stress levels was the most important risk factor for screw loosening (Tsuang et al., 2016; Pearson et al., 2017). Stress concentration on the bone-screw interfaces and screw rod systems will increase the risk of cancellous microdamage and resulting screw loosening (Nowak, 2019; Kanno et al., 2021). While discussing the risk factors for screw loosening, demographic characteristics are always assumed to be defined by some biomechanical pathogenesis. For instance, multiple studies have revealed that the incidence of screw loosening is high in senile patients with osteoporosis, which can be explained by the damage of bone-screw interface integration in vertebral bodies with low bone mineral density (BMD) (Bokov et al., 2019; Zou et al., 2020).

Clinical studies have shown that the mismatch between BEP and GB triggers complications, including nonunion and cage subsidence (Kim et al., 2012; Hu et al., 2021), and the mechanism of this phenomenon has been well explained. Specifically, biomechanical studies proved that the mismatch between BEP and GB changes the local load transmission pattern, and stress concentration can be observed on both the cranial and caudal sides of BEP and sub-BEP cancellous bone (Agarwal et al., 2013; Zhang et al., 2016). The risk of microdamage of bony structures and resulting cage subsidence should be increased (Mi et al., 2017; Lu and Lu, 2019). Meanwhile, the mismatch between BEP and GB can also lead to hypermobility of the surgical segment; resulting cage migration can also trigger cage subsidence, inhibit osteogenesis and increase the risk of nonunion (Agarwal et al., 2013; Zhang et al., 2016).

Studies proved that screw loosening was related to these complications, and insufficient anterior support was also reported as a risk factor for stress concentration in the bone-screw interfaces and resulting screw loosening (Kim et al., 2012; Bredow et al., 2016; Pearson et al., 2017; Hu et al., 2021). Considering that the mismatch between BEP and GB is a typical performance for insufficient anterior support, we hypothesize that it may also lead to the deterioration of stress levels and increase the risk of screw loosening. This study identifies whether the mismatch between BEP and GB will lead to local mechanical deterioration and resulting screw loosening from the perspective of radiographic observation and biomechanical research. This study collected imaging and demographic data from patients with single-segment OLIF fixed by the ALSR system. Biomechanical changes from OLIF models with different grades of contact sufficiency have been investigated in a calibrated and well-validated lumbosacral model. The published literature has not adequately clarified this issue.

In this study, we verified whether the mismatch between BEP and GB triggers a higher incidence of screw loosening and investigated the biomechanical effects of this phenomenon. Imaging data of OLIF patients fixed by ALSR have been retrospectively reviewed, and the biomechanical changes in ALSR and bone-screw interfaces have been computed in an anteriorly constructed numerical lumbo-sacral model. This study could provide theoretical guidance for understanding the screw loosening mechanism and optimizing the design of OLIF cages.

## Materials and Methods

### Review of Prospectively Collected Radiographic and Demographic Data

#### Patient Collection

The ethics committees of West China Hospital reviewed and approved the protocol of this study (2020-554). Informed consent was waived for this retrospective study. We retrospectively reviewed patients who underwent single segment OLIF with ALSR screw fixation from May 2017 to August 2019. The age, sex, and BMI of these patients were recorded. A senior spine surgeon performed all operations. Screw types and sizes were identical in these patients. All screws were placed in a single attempt and penetrated the contralateral cortex.

Patients who underwent single segment OLIF with ALSR screw fixation for lumbar degenerative diseases, including spinal stenosis, grade 1 and grade 2 degenerative spondylolisthesis, and lumbar disc herniation, were included in this study. The exclusion criteria were as follows: 1) Patients with a history of lumbar surgery; 2) Patients with primary or metastatic spinal tumors, lumbar tuberculosis, rheumatic immune diseases, and secondary osteoporosis caused by medication or other metabolic diseases; 3) Patients with grade 3 and grade 4 degenerative spondylolisthesis or spondylolysis; 4) Patients who underwent lumbar revision surgery within the clinical follow-up period of 12 months for complications other than screw loosening; 5) Patients who underwent intraoperative screw replacement.

#### Collection Radiographic Data

All patients underwent lumbar computational tomography (CT) three times in the imaging center of our hospital, including 1 week before, 1 week after, and 1 year after OLIF surgery (tube voltage: 120 kV) (Mikula et al., 2019; Xi et al., 2020; Zou et al., 2020). The CT scan settings were uniform in all enrolled patients. An experienced spine surgeon independently measured the following radiographic parameters. The interobserver and intraobserver reliability of these measured parameters was verified in 10 randomly selected patients. One week after the imaging measurement, the spine surgeon and a senior radiologist independently remeasured the imaging parameters of these selected patients.

**FIGURE 1:**
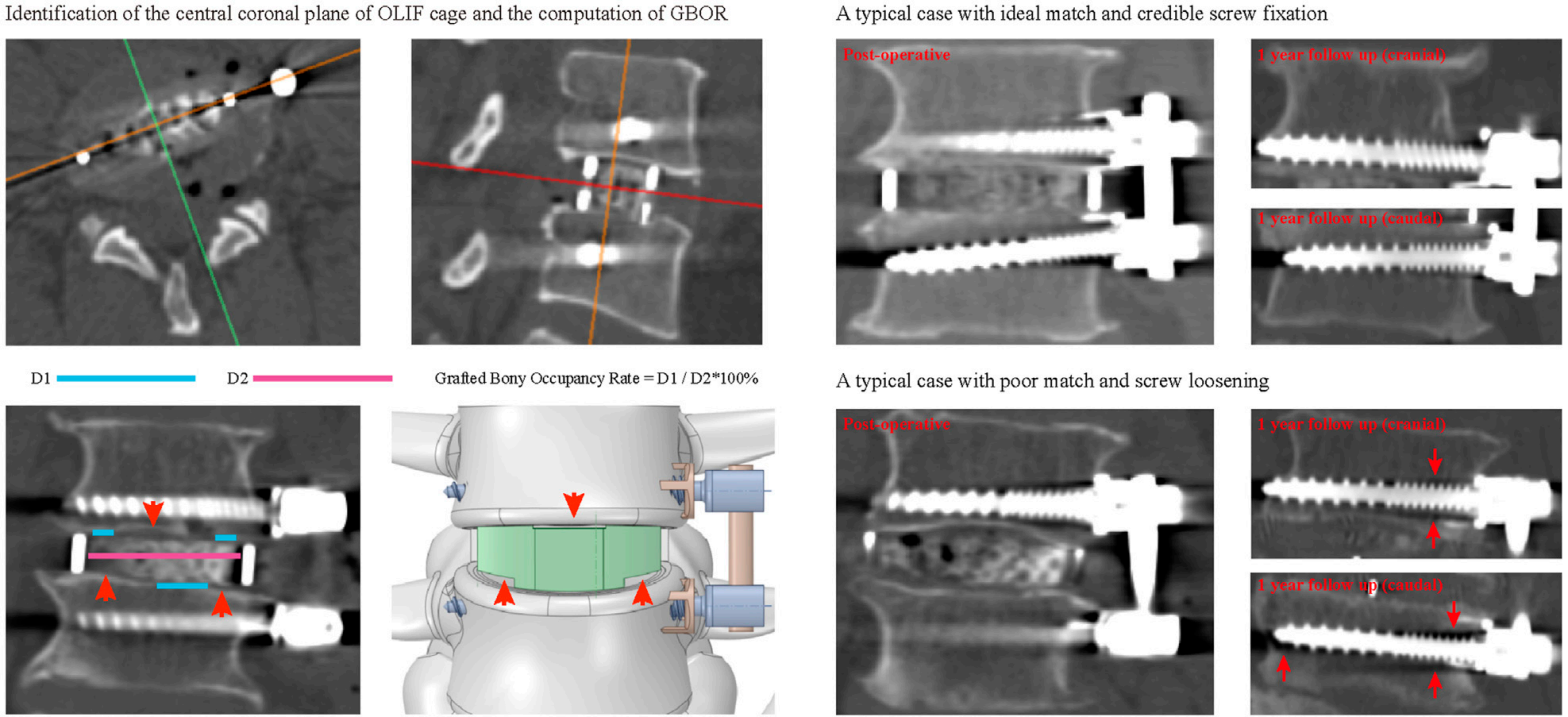
The schematic of the definition and surgical simulations of GBOR, and typical cases for different contact sufficiency and resulting screw fixation status.

The screw loosening status of the cranial and caudal vertebral bodies was identified separately. In the postoperative 1-year CT imaging data, vertebral bodies with ≥1 mm width radiolucent zones around the screw were defined as screw loosening (Bredow et al., 2016; Bokov et al., 2019; Zou et al., 2020). The BMD of these patients was identified by measuring their Hounsfield unit (HU) values. During HU measurement in vertebral bodies, the region of interest (ROI) was expanded to the largest within the cancellous bone but excluded other bony structures, such as cortical, BEP, and osteophytes (Schreiber et al., 2014; Xi et al., 2020; Zou et al., 2020). Values of HU were measured at the midsagittal plane, central transverse plane, transverse planes close to the superior and the inferior endplate separately, and the average value of these planes was set as the HU of the vertebral body (Pickhardt et al., 2013; Mikula et al., 2019; Xi et al., 2020; Zou et al., 2020). The sufficiency of contact between BEP and GB was quantified by calculating the grafted bony occupancy rate (GBOR) (Kim et al., 2012; Ushirozako et al., 2020). GBOR was measured in the cage’s central sagittal and coronal planes (rather than the vertebral body) in the postoperative CT imaging data (Figure 1).

#### Statistical Analyses

Radiographic and demographic indicators are presented as the mean ± standard deviation for continuous variables and number (percentage) for categorical variables. We conducted statistical analyses in SPSS software. The intraclass correlation efficiency (ICC) was computed to identify the repeatability of continuous variables (ICC ≥ 0.8 represents excellent reliability) (Zou et al., 2019; Zou et al., 2020). The kappa values were computed to determine the repeatability of screw loosening (kappa values of 0.41–0.60 indicated moderate reliability; 0.61 to 0.80, substantial agreement; and 0.81 to 1.00, excellent or almost perfect agreement) (Oetgen et al., 2008; Yue et al., 2008; Li et al., 2021b).

Statistical analyses for cranial and caudal side screw loosening were performed separately. When comparing the difference between different groups, the independent samples Student’s *t* test was used for continuous variables, and the chi-square test was used for the categorical variables. We performed binary logistic regression to identify independent risk factors for screw loosening. Univariate analyses of each potential risk factor were performed, and the variables that achieved a significance level of *p* < 0.1 were entered into multivariate analyses. Variables with *p* < 0.05 were considered independent risk factors in the multivariate analyses (Zhao et al., 2009; Park et al., 2017; Bagheri et al., 2019). A *p* value less than 0.05 indicated a significant difference.

### Numerical Surgical Simulations and Finite Element Analyses (FEA)

#### Construction of the Intact Finite Element (FE) Model

Our published studies have constructed and validated a biomimetic lumbosacral FE model (L3-S1). Bone structures of the FE model include cortical, cancellous, and BEPs. The cortical thickness was set as 0.8 mm, and the thickness and morphology parameters (i.e., concave angles and depths) of BEPs were defined separately based on anatomic studies (Li et al., 2021a; Li et al., 2021b). Nonbony components include the intervertebral disc (IVD) and facet cartilages. The IVD consists of the nucleus, annulus, and cartilage endplates (CEPs). The nucleus’s cross-sectional area accounted for 38% of the IVD (Li et al., 2021a; Li et al., 2021b). The outline of the BEP covers the entire IVD, and that of the CEP covers the nucleus and inner part of the annulus (Jacobs et al., 2014; DeLucca et al., 2016). Ligaments and facet capsules were defined as cable elements in the preprocessing process of FEA (Chuang et al., 2013; Dreischarf et al., 2014; Du et al., 2016; Li et al., 2019; Li et al., 2021a).

#### OLIF Simulations With Different Grades of Contact Sufficiency

The L4-L5 segment was selected to simulate oblique lumbar interbody fusion (OLIF) with ALSR screw fixation, and we performed surgical simulation according to a literature review and our surgical experience (Guo et al., 2020; Xi et al., 2020). In this process, lateral parts of the annulus, all of the nucleus, and CEPs were removed, and a polyether-ether-ketone (PEEK) OLIF cage (18 mm width and 50 mm length) filled with grafted bone was inserted into the interbody space. The lordotic angle and disc height of the postoperative models were identical to those of the intact model to eliminate the mechanical effects of these parameters (Kim et al., 2012; Wang et al., 2019; Guo et al., 2020).

Three different grades of contact sufficiency between the BEP and GB (including ideal, acceptable, and poor) were simulated by changing the GBOR in the coronal plane. The ideal contact was defined completely match between BEP and GB, GBOR in acceptable and poor contact models were defined as 80 and 60%, respectively. Based on the review of radiographic data, the mismatch on the superior side was mainly in the central region, while that on the inferior side was in the peripheral region. By combining different contact sufficiency grades between the GB and superior and inferior BEPs, five different OLIF models were constructed (Table 1; Figures 1, 5).

**TABLE 1:** Construction of numerical models with different grades of contact sufficiency.

	Model1	Model2	Model3	Model4	Model5
	Ideal	Ideal	Poor	Acceptable	Poor
Cranial					
	GBOR = 100%	GBOR = 100%	GBOR = 60%	GBOR = 80%	GBOR = 60%
	Ideal	Poor	Ideal	Acceptable	Poor
Caudal					
	GBOR = 100%	GBOR = 60%	GBOR = 100%	GBOR = 80%	GBOR = 60%

During the simulation of ALSR screw fixation, two titanium alloy (TI) screws were inserted into the L4-L5 vertebral bodies and penetrated the contralateral cortex. The axes of the screws in the transverse plane were parallel to the OLIF cage, whereas those in the coronal plane were parallel to the BEPs (Guo et al., 2020; Xie et al., 2020). Screw threads were preserved, and the screw compaction effect was simulated by adjusting the material property of cancellous around the thread (Hsu et al., 2005; Matsukawa et al., 2016). The connection between the screw tulip, the nut, and the spacer was simplified to increase the computational efficiency.

#### Boundary and Loading Conditions

Finite element analyses in this study were performed in the “Ansys workbench 2020 r2 academic”. Hybrid elements (e.g., tetrahedron and hexahedron elements) with different sizes were set in different components of the FE model. Mesh refinement was set in structures with low thickness and large deformation (e.g., BEP, facet cartilage, and posterior parts of the annulus) (Kim et al., 2010; Chuang et al., 2013; Dreischarf et al., 2014; Kang et al., 2017). The degrees of freedom of S1 inferior surfaces were fixed entirely. Different directional moments were applied on the superior BEP of L3 (DeLucca et al., 2016; Li et al., 2021a). Numerical simulations computed under flexion, extension, left and right bending, and axial rotation loading conditions (Figure 3). In the definition of material properties (Table 2), cortical and cancellous bone were defined by anisotropic law (Ferguson and Steffen, 2003; Morgan et al., 2003; Tsouknidas et al., 2015). The annulus was assumed to be hypoelastic material, and the nucleus was set as a semifluid incompressible material (Wu and Yao, 1976; Kim et al., 2010). The material properties of the surgical instrumented structure (i.e., PEEK and TI) were defined by isotropic law; the elastic modulus of the GB was calculated based on the HU values measured in the postoperative CT scan. By defining the friction coefficients between different contact surfaces, stress levels immediately after operation were computed (Chuang et al., 2012; Hsieh et al., 2017; Kang et al., 2017). The contact between facet cartilages was set as frictionless, the frictional coefficient between BEP and GB was 0.46, and that between BEP and cage and screw-cancellous interfaces was 0.2 (Lu and Lu, 2019; Rastegar et al., 2020).

**TABLE 2:** Material properties of FE models’ components.

Components	Elastic modulus (MPa)	Poisson’s ratio	Cross-section (mm^2^)	References
Cortical	E_xx_ = 11,300	V_xy_ = 0.484		Ferguson and Steffen (2003); Tsouknidas et al. (2015)
	E_yy_ = 11,300			
	E_zz_ = 22,000	V_yz_ = 0.203		
	G_xy_ = 3,800			
	G_yz_ = 5,400	V_xz_ = 0.203		
	G_xz_ = 5,400			
Cancellous	E_xx_ = 140	V_xy_ = 0.45		Morgan et al. (2003); Tsouknidas et al. (2015)
	E_yy_ = 140			
	E_zz_ = 200	V_yz_ = 0.315		
	G_xy_ = 48.3			
	G_yz_ = 48.3	V_xz_ = 0.315		
	G_xz_ = 48.3			
Bony endplates	12,000	0.3		Kang et al. (2017); Li et al. (2019)
Annulus	Hypoelastic material			Wu and Yao (1976); Kim et al. (2010)
Nucleus	1	0.49		Chuang et al. (2013); Qasim et al. (2014)
Cartilage endplates	10	0.4		Li et al. (2019); Li et al. (2021)
Anterior longitudinal ligaments	Calibrated load-deformation curved under different loading conditions	0.3	60	Du et al. (2016); Li et al. (2021)
Posterior longitudinal ligaments	Calibrated load-deformation curved under different loading conditions	0.3	21	Du et al. (2016); Li et al. (2021)
Ligamentum flavum	Calibrated load-deformation curved under different loading conditions	0.3	60	Du et al. (2016); Li et al. (2021)
Interspinous ligaments	Calibrated load-deformation curved under different loading conditions	0.3	40	Du et al. (2016); Li et al. (2021)
Supraspinous ligaments	Calibrated load-deformation curved under different loading conditions	0.3	30	Du et al. (2016); Li et al. (2021)
Intertransverse ligaments	Calibrated load-deformation curved under different loading conditions	0.3	10	Du et al. (2016); Li et al. (2021)
Capsular	7.5 (\25%)	0.3	67.5	Chuang et al. (2013); Li et al. (2019)
	32.9 ([25%)			
PEEK OLIF Cage	3,500	0.3		Hsieh et al. (2017); Kang et al. (2017)
Titanium alloy screw	110,000	0.3		Hsieh et al. (2017); Kang et al. (2017)

#### Model Calibration and Validation

The stiffness of ligaments was seen as a calibrated indicator. By repeatedly computing the range of motions (ROMs) in the L4-L5 segment and adjusting ligament stiffness, the differences between computed ROMs and measured values from widely cited *in vitro* studies could be reduced (Schmidt et al., 2007a; Schmidt et al., 2007b; Du et al., 2016; Li et al., 2021a). As a result, current FE models could better represent real stress levels by model calibration. We performed a mesh convergence test on the calibrated intact model by evaluating the change in intradiscal pressure (IDP) with different mesh sizes. The model was considered converged if the change in the computed IDP was less than 3% (Ottardi et al., 2016; Fan et al., 2021). The computed ROM, IDP, disc compression (DC), and facet contact force (FCF) were compared with *in vitro* measured values in the multi-indicator model validation process (Wilson et al., 2006; Renner et al., 2007; Schilling et al., 2011).

## Results

### Retrospectively Study of Prospectively Collected Data

#### Patient Collection and Screw Loosening Rates

A total of 56 patients (30 males and 26 females) with an average age of 56.57 ± 11.96 years treated by single segment OLIF with ALSR screw fixation were recorded. The interobserver and intraobserver results during the judgment of screw loosening were substantial, with Kappa values of 0.778 and 0.759, respectively. The reliability of continuous variable measurement was excellent, with ICCs of 0.894 and 0.862, respectively (Table 3). The overall incidence rate of screw loosening was 35.71% (40/112), and the screw loosening rate of the vertebral body on the cranial side was 42.86% (24/56), which was significantly higher than that of the caudal vertebral body, which was 28.57% (16/56, *p* = 0.002). The cranial side’s GBOR was significantly lower than that of the caudal side in coronal and sagittal planes (*p* = 0.009), and there were no significant differences in HU between cranial and caudal vertebral bodies (*p* = 0.519).

**TABLE 3:** Validation of measured values repeatability.

	Interobserver	Intraobserver
ICCs of continuous variables	0.894	0.862
Kappa values of union status	0.778	0.759

#### Identification of Independent Risk Factors for Screw Loosening

**FIGURE 2:**
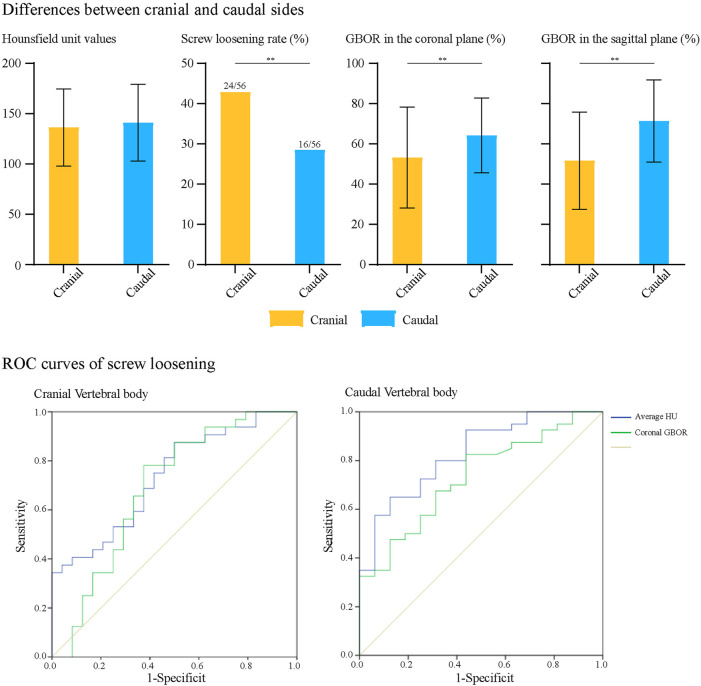
ROC curves for cranial and caudal side screw loosening.

The age of patients with cranial side screw loosening was significantly higher (*p* = 0.033) and had significantly lower coronal plane GBOR and HU than those without screw loosening. The *p* value of HU was 0.003, and that of GBOR was 0.013. Based on the computational results of univariate logistic regression analyses, these three indicators were also entered into the multivariate analysis to identify independent risk factors. The results showed that reducing HU and coronal plane GBOR were independent risk factors for screw loosening on the cranial side (Tables 4, 5). The *p* value of HU was 0.019, and that of GBOR was 0.043. In regard to caudal side screw loosening, differences in GBOR on the coronal plane and HU were significant in the screw loosening and nonloosening groups; the *p* value of HU was 0.000, and that of GBOR was 0.005. Based on univariate logistic regression analyses, HU and GBOR in coronal and sagittal planes were entered into the multivariate analysis. Consistent with the cranial vertebral body, reduced HU and coronal plane GBOR were also independent risk factors for screw loosening in the caudal vertebral body (Figure 2 and Table 6). The *p* value of HU was 0.001, and that of GBOR was 0.023.

**TABLE 4:** Logistic regression analysis of the cranial screw loosening.

	OR	95% CI		*p*
Univariate analysis				
Gender	2.333	0.791	6.885	0.125
Age	1.053	1.003	1.106	0.039^a^
BMI	0.972	0.83	1.138	0.723
Average HU	0.976	0.959	0.993	0.005^a^
GBOR (coronal plane)	0.971	0.949	0.995	0.017^a^
GBOR (Sagittal plane)	0.988	0.966	1.011	0.3
Multivariate analyses				
Age	1.028	0.971	1.089	0.341
Average HU	0.978	0.96	0.996	0.019^b^
GBOR (coronal plane)	0.973	0.948	0.999	0.043^b^

a Variables that achieved a significance level of *p* < 0.1 in the univariate analysis.

b Statistical significance in the multivariate regression analysis (*p*＜0.05).

**TABLE 5:** Logistic regression analysis of the caudal screw loosening.

	OR	95% CI		*p*
Univariate analysis				
Gender	1.739	0.54	5.604	0.354
Age	1.042	0.99	1.097	0.117
BMI	0.985	0.828	1.17	0.86
Average HU	0.957	0.933	0.982	0.001^a^
GBOR (coronal plane)	0.951	0.915	0.988	0.01^a^
GBOR (Sagittal plane)	0.974	0.946	1.002	0.071^a^
Multivariate analyses				
Average HU	0.953	0.927	0.98	0.001^b^
GBOR (coronal plane)	0.94	0.89	0.992	0.023^b^
GBOR (Sagittal plane)	0.996	0.956	1.038	0.852

a Variables that achieved a significance level of *p* < 0.1 in the univariate analysis.

b Statistical significance in the multivariate regression analysis (*p*＜0.05).

**TABLE 6:** The cut-off value, sensitivity and specificity of four measurement methods for predicting screw loosening.

	Cut-off value	Sensitivity	Specificity	AUC
Cranial vertebral body				
Average HU	105.56	0.875	0.5	0.733
Coronal plane’s GBOR (%)	55.27	0.656	0.6.67	0.686
Caudal vertebral body				
Average HU	107.3	0.925	0.562	0.83
Coronal plane’s GBOR (%)	60.97	0.675	0.687	0.732

#### Parameter Prediction Values for Screw Loosening

We performed ROC curve analyses to assess the predictive value of HU and coronal plane GBOR; the results are summarized in Figure 2 and Table 6. Consistent with logistic regression analyses, HU values of vertebral bodies had the highest predictive ability. The AUCs of HU in the cranial and caudal vertebral bodies were 0.733 and 0.830, and those of the coronal plane’s GBOR were 0.686 and 0.732, respectively.

### Numerical Mechanical Surgical Simulations

#### Multi-Indicator Model Validation

**FIGURE 3:**
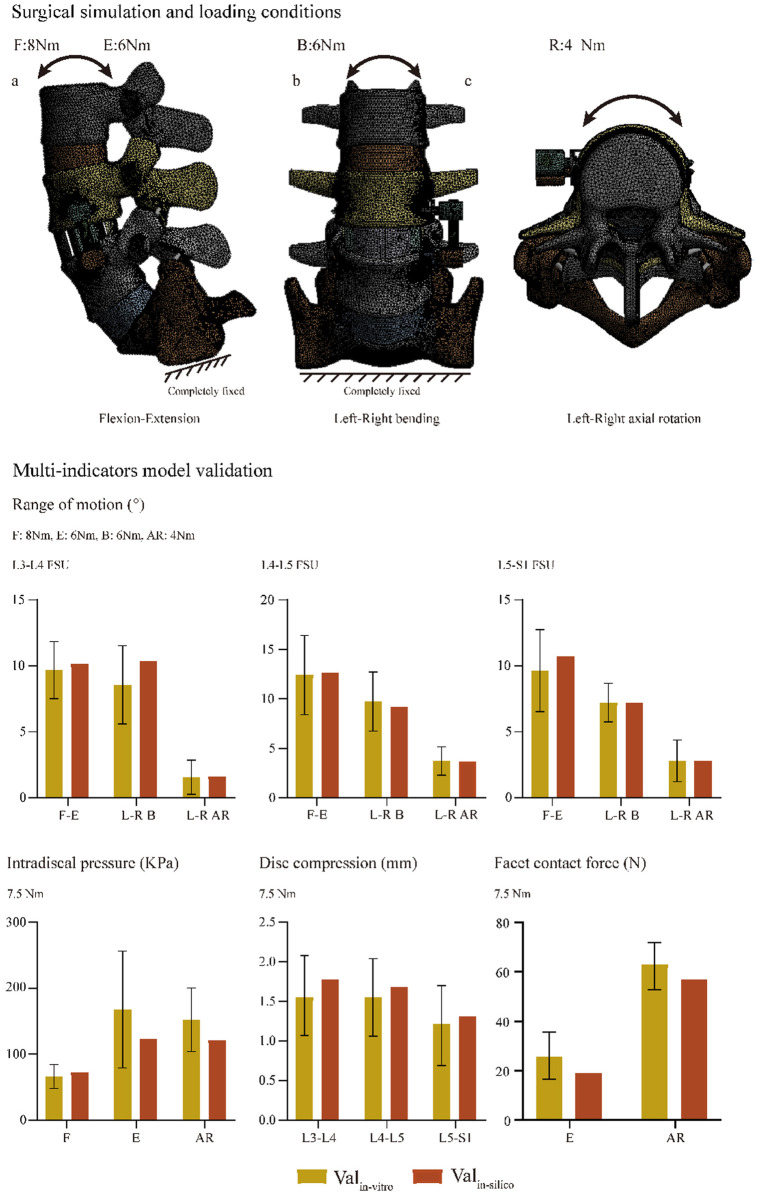
Surgical simulations and multi-indicator model validations.

Biomechanical indicators computed by the calibrated intact model were within ±1 standard deviation of the average values measured by *in vitro* studies. Thus, we believe that biomechanical changes identified by current FE models make good representations of actual stress levels (Figure 3).

#### Biomechanical Changes Caused by the Change in GBOR in the Coronal Plane

**FIGURE 4:**
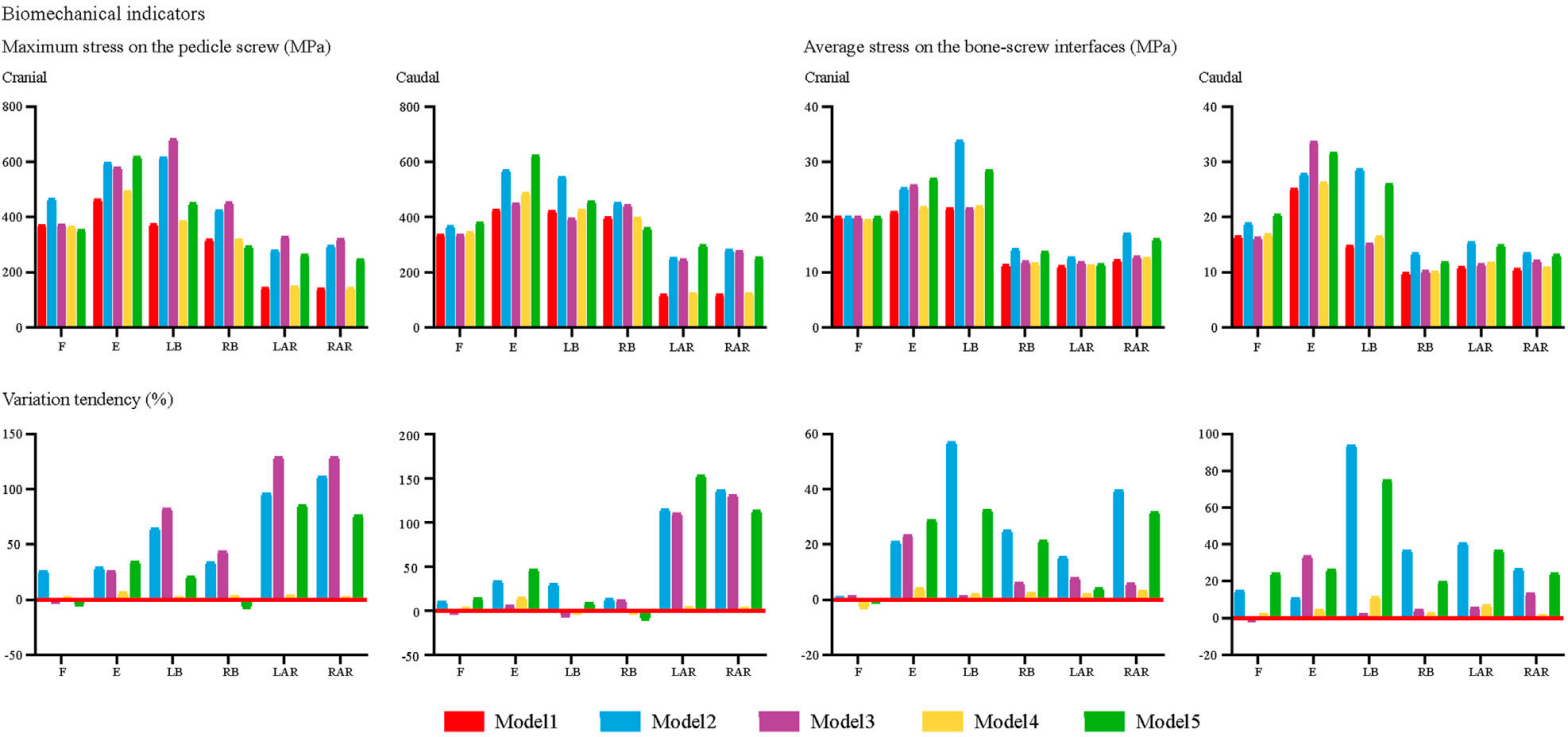
Changes in biomechanical indicators.

**FIGURE 5:**
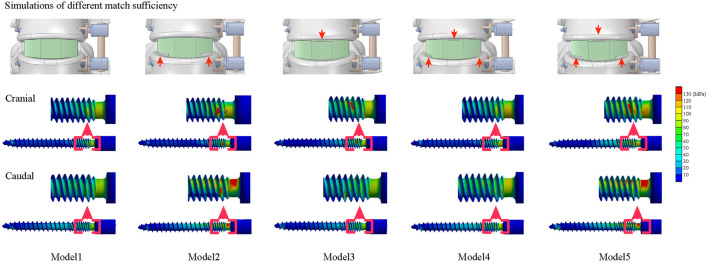
Different match sufficiency and nephograms of screws.

We computed the maximum von Mises stress of both cranial and caudal screws and the average stress of corresponding bone-screw interfaces to investigate the risk of screw loosening biomechanically (Ambati et al., 2015; Matsukawa et al., 2016; Fletcher et al., 2019; Kim et al., 2020); changes in computed biomechanical indicators can well explain the result from our review of radiographic data. Consistent with published studies, stress concentration can be observed in the screw head of both cranial and caudal screws (Chao et al., 2008; Amaritsakul et al., 2014). Compared to the model with ideal contact sufficiency, a slight stress concentration of the screw and corresponding bone-screw interfaces can be recorded with the acceptable (80%) contact model. In contrast, the stress values of the poor contact models dramatically increased under almost all body positions, especially under the left lateral bending and two-sided axial rotation loading conditions (Figures 4, 5).

## Discussion

Stress concentration of the ALSR fixation system and bone-screw interfaces and the resulting loss of bone-screw integration are primary causes of screw loosening. Therefore, biomechanical changes should provide reasonable explanations for screw loosening-related clinically observed factors and provide theoretical references for optimizing treatment strategies. Taking the correlation between BMD reduction and the increased risk of screw loosening as an example: Consistent with the current study, clinical studies repeatedly proved that osteoporosis is an independent risk factor for predicting screw loosening (Bredow et al., 2016; Bokov et al., 2019). Biomechanical studies, including numerical simulations and *in vitro* mechanical tests, have also repeatedly proven that BMD reduction would lead to the deterioration of screw-bone integration and the resulting reduction of screw pull-out and fixation strength (Ohtori et al., 2013; Weidling et al., 2020; Zou et al., 2020). These findings have contributed to the updating of treatment principles. Regular anti-osteoporosis therapy has been promoted in osteoporosis patients requiring internal spinal fixation as an effective method to reduce the risk of screw loosening (Ohtori et al., 2013; Mikula et al., 2019).

Poor matches between BEP and GB biomechanically trigger complications. Clinical follow-up studies proved that a poor match would increase the risk of cage subsidence and nonunions (Kim et al., 2012; Hu et al., 2021); biomechanical studies presented that the poor match between BEP and GB triggers BEP stress concentration and hypermobility of the surgical segment (Agarwal et al., 2013; Zhang et al., 2016). These studies conclude that optimizing cage design based on the morphological difference of BEPs could reduce the risk of these complications by optimizing the local stress level. However, no studies have identified the effect of poor contact between the BEP and GB on the incidence of screw loosening. In patients fixed by the pedicle screw system, poor anterior column support was an essential trigger for biomechanical deterioration in the bone-screw interfaces, and the mismatch between BEP and GB can be seen as a typical instance of “poor anterior support” (Bredow et al., 2016; Pearson et al., 2017; Hu et al., 2021). Therefore, we proposed and verified the hypothesis that the mismatch between the BEP and GB may also increase the risk of screw loosening for ALSR fixation biomechanically. We believe this study’s most significant innovation effectively combines radiographic observation with numerical simulations compared with the same type of published studies. In these studies, clinical phenomena and biomechanical effects have been investigated separately (Kim et al., 2012; Agarwal et al., 2013; Zhang et al., 2016; Hu et al., 2021). In contrast, the current study constructs operative models to explore the biomechanical effects of clinically independent risk factors. We believe that biomechanical parameters computed by these FE models could provide credible theoretical guidance for optimizing spinal instrumented devices (i.e., the OLIF cage).

Radiographic observations showed that reducing the GBOR in the coronal plane was also an independent risk factor for screw loosening in the cranial and caudal vertebral bodies. Biomechanical changes in screws and corresponding bone-screw interfaces in models with different match sufficiency have been computed in numerical surgical simulations (Ambati et al., 2015; Matsukawa et al., 2015; Fletcher et al., 2019; Kim et al., 2020). The effectiveness of these indicators in predicting the risk of screw loosening has been well demonstrated in previous biomechanical studies. Corresponding to the review of radiographic data, the poor match between BEP and GB increases the load transmitted by the ALSR system, and stress concentration on the bone-screw interfaces will lead to the microdamage of cancellous bone and resulting screw loosening. Therefore, the increase in match sufficiency by optimizing cage design should be significant for reducing screw loosening risk.

*In vitro* mechanical tests on fresh specimens, the “gold standard” of biomechanical studies, were not performed in this study for the following reasons. When using a particular type of OLIF cage, the contact sufficiency between the BEP and GB mainly depends on the morphology parameters of the BEPs in different specimens. Considering that fresh specimens are very scarce and it is difficult to obtain sufficient specimens with different morphology parameters, it is unenforceable to perform the biomechanical test of this study by *in vitro* fresh specimen testing. Meanwhile, the mechanical effects of confounding factors (e.g., differences in BMD in different specimens) could not be excluded effectively in a small sample size study (Bredow et al., 2016; Bokov et al., 2019). In addition, the purpose of this study is to provide biomechanical references for the necessity of manufacturing OLIF cages that can match different BEP morphology parameters. Cage manufacturing is based on the results of mechanical tests, not the other way around. It is challenging to produce cages with different outlines to achieve different degrees of contact sufficiency with limited fresh specimens. Additionally, it is challenging to directly insert stress sensors into bone-screw interfaces. As a result, in fresh specimen mechanical tests, cancellous stress distribution can only be inferred by indirect measured indicators (e.g., displacement of the screw fixation system and deformation of vertebral bodies) (Nowak, 2019; Kanno et al., 2021).

In contrast, we believe FEA is more suitable for investigating the mechanical effects of contact sufficiency on the risk of screw loosening. In this study, surgical simulations were performed in a single intact model, and only the coronal plane’s GBOR was adjusted in different FE models. This mechanical testing strategy can independently analyze the risk factor obtained from clinical observation, exclude the interference of other confounding factors, and obtain a more reliable conclusion (Dreischarf et al., 2014; Zhang and Gong, 2020). More significantly, details of the stress distribution on the bone-screw interfaces can be directly measured in FEA models (Xu et al., 2019; Takenaka et al., 2020). Without worrying about the difficulty of model sourcing, we can demonstrate the biomechanical effects of different match degrees between BEP and GB in the FEA study. By adjusting the cage outline to achieve different matching degrees, we can directly observe its biomechanical effects on the risk of screw loosening, which can provide a reliable reference for optimizing cage design.

Indeed, this study still has inherent limitations. Although screw loosening commonly occurred in the early stage (6 months) after spinal fixation, the variation tendency was evident at the 1-year clinical follow-up. As a radiographic review with limited sample sizes and a short follow-up period, we still should admit that the results of this study cannot be generalized to long-term clinical outcomes. In addition, in FEA, we did not identify the mechanical effects of the sagittal plane’s GBOR. Although this factor is not an independent risk factor for screw loosening, it should be considered in our subsequent studies to evaluate the interaction of coronal and sagittal plane GBOR and their mechanical effects on the risk of screw loosening. Additionally, although this was the common method for the same type studies (Chuang et al., 2012; Kim et al., 2015; Hsieh et al., 2017; Kang et al., 2017; Li et al., 2021a), the multi-indicator model validation was only performed in the preoperative intact spine. The stress distribution of the ALSR system and bone-screw interfaces were computed in postoperative models without validation.

## Conclusion

Based on the radiographic review and numerical surgical simulations, we can conclude that the mismatch between the BEP and GB will lead to stress concentration on the ALSR and bone-screw interfaces and increase the resulting risk of screw loosening. Cage design modification is of great significance for reducing screw loosening risk biomechanically.

## Data Availability

The original contributions presented in the study are included in the article/Supplementary Material, further inquiries can be directed to the corresponding authors.

## Abbreviations

ALSR, anterior lateral single rod; AUC, area under the curve; BEP, bony endplate; BMD, bone mineral density; CEP, cartilage endplate; CT, computational tomography; DC, disc compression; FCF, facet contact force; FEA, finite element analysis; GB, grafted bone; GBOR, grafted bony occupancy rate; HU, Hounsfield units; ICC, intraclass correlation efficiency; IDP, intradiscal pressure; OLIF, oblique lumbar interbody fusion; PEEK, polyether ether ketone; ROC, receiver operating characteristics; ROI, region of interest; ROM, range of motion; TI, titanium.
